# Supplementary material for: Effectiveness of health promoting schools: A comparative health profile assessment of higher as compared to low accredited schools in Chandigarh, Union Territory of North India
Source: PLoS One. 2022 Sep 30;17(9):e0270811. doi: 10.1371/journal.pone.0270811 (PMC9524632; doi:10.1371/journal.pone.0270811)
Supplement: Attachment — Submitted filename: Response to Reviewers.docx [file pone.0270811.s002.docx]

|  | Comments |  |
| --- | --- | --- |
| Reviewer 1 | | |
|  | Study on Effectiveness of Health Promoting School (HPS) is very much needed. I commend the authors for the dedicated effort to gain new knowledge to evidence of HPS effectiveness. However, more work is needed for publication. | Thanks for appreciation |
|  | There should be more description of the accreditation of different levels of awards and the process of validation. | Reference of previous published study was already given. For further description Figure-1 is added in the manuscript. This is to inform that development and validation of standards published previously by the author.  Thakur, Jarnail; Sharma, Deepak; Jaswal, Nidhi; Bharti, Bhavneet; Grover, Ashoo; Thind, Paramjyoti (2014). *Developing and implementing an accreditation system for health promoting schools in Northern India: a cross-sectional study. BMC Public Health, 14(1), 1314–.* doi:10.1186/1471-2458-14-1314 |
|  | Comparison of basic characteristics of schools with different levels of award and non-award schools should be performed to analyse any difference at baseline. Those would be confounding factors. | Thanks for comment.  Our primary objective was to assess and classify all **private and government** schools located in a northern city of India for accreditation as health promoting schools.  Baseline characteristics of Govt and private schools were compared, and no difference was found. |
|  | The authors need to justify the categorisation between higher accreditation schools (Gold and Silver- 1+9=10) and lower accreditation schools (Bronze or below bronze-24+152=176). The standard below bronze would be variable and the scientific basis of putting bronze and below bronze in same category needs to be justified. | We appreciate this comment.  The objective of the study was to compare health profile of higher (Platinum, Gold, Silver) and lower (Bronze) accredited schools. On conducting the baseline study, it was found that majority of the schools were in the below bronze(N=152) category. The basic characteristics (student teacher ratio, toilet ratios, water consumption person per day) of bronze and below bronze schools were analysed and showed no difference. An expert group meeting was conducted to consider bronze level and below bronze categorised as lower accredited schools based on the comparison of basic characteristics. |
|  | Health profile of students from 8 higher accredition schools with 754 students were compared with 700 students from lower acrreditation ? numbers of schools. There is no description of sampling methods and the rationale behind. There were 152 lower accreditiation schools and this reference group can have larger sample size. If all schools with award are categorised as accedition schools, there will be students from 34 schools comparing with students from 152 non-accredition schools, this would allow better analysis of effect size. The Hong Kong studies published ib 2006 and 2008 cited by the authors categories schools into award and non-award groups. | For comparison between higher accredited and lower categorised schools, a sample size of 1400 was calculated by considering difference in prevalence of anaemia among rural and urban school going children (14.16% vs 25.4%) with power of 80% and a design effect of 1.5(Ref). A multistage random sampling was performed. The first stage consisted of random selection of 8 schools from the higher and lower accredited the schools. Letters of invitation were sent to each selected school. In the second stage, we randomly selected one section of classes (7 and 8) from each selected school with an inclusion criterion of a student studying in the school for the last two years. In Chandigarh, one section of a class is comprised of 40 to 50 students and there are 4-5 sections of a particular class. |
|  | Apart from oberall analysisi of pre and post interventions, authors can conduct separate pre and post interventions among accreditation schools and non-accreditiation schools. For comparison of health profiles, those factors. showing statistical signifiance, most of them only demonstrated less than 10% difference and many only around 5%. Authors would consider re-analysis with bronze schools to be included under acceditiation schools comparing with non-accreditation schools. If data available, should include all students from those schools and it will give rise to much larger sample size. | Noted.  It was not under scope of this paper.  Moreover, comparison of accredited and non-accredited is not possible, as all schools were assessed for accreditation  The current paper has the objective to assess and classify all private and government schools located in a northern city of India for accreditation as health promoting schools. Hence, the results are presented accordingly. |
|  | Multiple logisitic regression can be considered to analyse the factor(s) including accreditation status together with other independent variables predicting particular health outcomes. This will allow controlling the confounding factors. Those components under different domains showing statistical difference from pre and post analysis would also be included as independent variables to analyse which component(s) is independent predictor for better health profile. | Same as above |
|  | The authors should draw on more recent literature on HPS to strenghten the discussuon section. The speical issue of Health Promotion International on HPS in 2017 and other recent papers. | Done |

| Reviewer 2 |  |
| --- | --- |
| This is a pre-post multicomponent and multilevel health promotion school accreditation intervention study of both government and private secondary schools in Northern India. The aim was to compare the higher accredited schools versus lower accredited schools for health profile assessment outcomes. The authors showed an association between higher accreditation levels and time period (table 2), increase in two of eight accreditation domains associated with the intervention (table 3), higher hygiene levels and protective factors with higher accredited schools (table 4), and mental health improvements but poorer sexual health awareness associated with higher accredited schools (table 5). To improve the manuscript readability, the paper needs to be edited for conciseness and follow a more structured approach. It is not necessary to quote percentage to 2 decimal places in Tables-1 decimal place is sufficient. Be consistent with number of decimal places for P values throughout manuscript. | All values mentioned to 1 decimal place and p values mentioned to 2 decimal places |
| Title needs to be more informative. Possible suggestion “Association between health promotion school accreditation levels and health profile assessment over time in Northern India.” The use of effectiveness implies randomization. | We would like to retain the original title as quasi experimental study |
| Ethical statement. This need to be completed. Please specify name of ethics committee, reference number and date of approval. Also include these details in line 156. | Done |
| Introduction. Lines 83 to 105. This is a long paragraph  Suggest paragraph break at line 93 to improve readability. | Done |
| Consider a subheading ‘Intervention’ so it is clear what it is before line 116. | Done |
| Line 121. Bolded ‘Manual’. Is this subheading or part of a sentence? | Subheading made |
| Line 128. Bolded ‘Key school-based intervention’. Is this subheading or part of a sentence? | Subheading made |
| Line 131. Social media does not need to be bolded. | Done |
| Line 133. Bolded ‘Orientation and reorientation’ subheading or sentence? | Bolding removed |
| Line 141. Bolded ‘The health profile assessment’ subheading or sentence? | Bolding removed |
| Line 145-147. Specify exactly how schools were randomly selected. | 8 schools mentioned. Diagram included |
| Write out in full abbreviations ‘CBSE’ (line 149), indicate what SHAALA SIDDHI in Table 3 means with footnote. | Done |
| Line 149 Include reference | Done |
| Line 161. Specify version, company and city for SPSS and Epiinfo. | Done |
| State significant level P<0.05 and 2-sided significance in data analysis. | Done |
| Text in Results is too long. Much of it is repeating the information in the Tables. Consider adding a flow diagram of schools, like STROBE, to help reader understand the study design and data analysis. | Strobe flow diagram added |
| Table 1. Median (IQR) for sex characteristics are not clear. Is this number of girls and boys in each school? | Yes, IQR for both boys and girls mentioned |
| Table 2. Column for preintervention private school column does not add up to 100%. Consider conducting a Cochrane-Armitage test for trend for total pre and post intervention accreditation level, which would be significant to show that overall accreditation levels changed over time. %age change should be rewritten to Change with a footnote to indicate percentage; consider 95% CI around percentage change. | Table 2 correction done.  Thanks for this suggestion and Cochrane Armitage test cannot be performed for total pre and post accreditation level as it was not under scope of this paper.The primary objective is to compare the Govt and private schools |
| Table 3. Title Score ≥3 is unclear to readers. Please explain. | As per previous paper published.  Thakur, Jarnail; Sharma, Deepak; Jaswal, Nidhi; Bharti, Bhavneet; Grover, Ashoo; Thind, Paramjyoti (2014). *Developing and implementing an accreditation system for health promoting schools in Northern India: a cross-sectional study. BMC Public Health, 14(1), 1314–.* doi:10.1186/1471-2458-14-1314 |
| Table 4. Check all P value, especially for BMI first row and third row, should this be marginally significant? | It was rechecked and found the same |
| The discussion was not concise and structured. First paragraph should highlight main results as indicated in general comments above. Then following paragraphs should discussed each of the main results in sequence with Table order in comparison with other published studies. There should be a study limitation paragraph. | Done |
