# Supplementary material for: Effectiveness of health promoting schools: A comparative health profile assessment of higher as compared to low accredited schools in Chandigarh, Union Territory of North India
Source: PLoS One. 2022 Sep 30;17(9):e0270811. doi: 10.1371/journal.pone.0270811 (PMC9524632; doi:10.1371/journal.pone.0270811)
Supplement: Attachment — Submitted filename: Reviewers_comments.docx [file pone.0270811.s003.docx]

**Response to Reviewers**

| Have the authors made all data underlying the findings in their manuscript fully available?  The [PLOS Data policy](http://www.plosone.org/static/policies.action#sharing) requires authors to make all data underlying the findings described in their manuscript fully available without restriction, with rare exception (please refer to the Data Availability Statement in the manuscript PDF file). The data should be provided as part of the manuscript or its supporting information, or deposited to a public repository. For example, in addition to summary statistics, the data points behind means, medians and variance measures should be available. If there are restrictions on publicly sharing data—e.g. participant privacy or use of data from a third party—those must be specified.  Reviewer #2: No | Data cannot be shared publicly to maintain the privacy and confidentiality of the participating private and government schools. Data request may be sent to PGIMER institute committee ( [iecpgi@gmail.com](mailto:iecpgi@gmail.com)) for researchers who meet the criteria for access to confidential data. |
| --- | --- |
| Reviewer #2: The revised manuscript reads much better. Just further minor amendments to be made: 1. Move limitation of the study to before conclusion section 2. Provide exact grant number of the government funding agency grant you obtained for completeness 3. Relabel 'Figure 2 Strobes flow diagram' to 'Figure 2 Participating Schools Flow Diagram' | We appreciate the comments of the reviewer and thankful for valuable inputs.   1. Limitation shifted before conclusion section. 2. Grant no. File no DHR/Plan Scheme/GIA/16/2012,   IRIS Cell (2012-27290 dated 24.01.2013)   3. Done. Figure-2 relabelled. |
|  |  |
